# High-LET Carbon and Iron Ions Elicit a Prolonged and Amplified p53 Signaling and Inflammatory Response Compared to low-LET X-Rays in Human Peripheral Blood Mononuclear Cells

**DOI:** 10.3389/fonc.2021.768493

**Published:** 2021-11-23

**Authors:** Ellina Macaeva, Kevin Tabury, Arlette Michaux, Ann Janssen, Nicole Averbeck, Marjan Moreels, Winnok H. De Vos, Sarah Baatout, Roel Quintens

**Affiliations:** ^1^ Radiobiology Unit, Studiecentrum voor kernenergie - Centre d'étude de l'énergie nucléaire (SCK CEN), Mol, Belgium; ^2^ Department of Molecular Biotechnology, Ghent University, Ghent, Belgium; ^3^ Department of Oncology, KU Leuven, Leuven, Belgium; ^4^ Department of Biomedical Engineering, University of South Carolina, Columbia, SC, United States; ^5^ Department of Biophysics, GSI Helmholtzzentrum für Schwerionenforschung, Darmstadt, Germany; ^6^ Department of Veterinary Sciences, University of Antwerp, Antwerp, Belgium

**Keywords:** ionizing radiation, gene expression, DNA damage, X-rays, heavy ions, p53, alternative splicing, immunity

## Abstract

Understanding the differences in biological response to photon and particle radiation is important for optimal exploitation of particle therapy for cancer patients, as well as for the adequate application of radiation protection measures for astronauts. To address this need, we compared the transcriptional profiles of isolated peripheral blood mononuclear cells 8 h after exposure to 1 Gy of X-rays, carbon ions or iron ions with those of non-irradiated cells using microarray technology. All genes that were found differentially expressed in response to either radiation type were up-regulated and predominantly controlled by p53. Quantitative PCR of selected genes revealed a significantly higher up-regulation 24 h after exposure to heavy ions as compared to X-rays, indicating their prolonged activation. This coincided with increased residual DNA damage as evidenced by quantitative γH2AX foci analysis. Furthermore, despite the converging p53 signature between radiation types, specific gene sets related to the immune response were significantly enriched in up-regulated genes following irradiation with heavy ions. In addition, irradiation, and in particular exposure to carbon ions, promoted transcript variation. Differences in basal and iron ion exposure-induced expression of DNA repair genes allowed the identification of a donor with distinct DNA repair profile. This suggests that gene signatures may serve as a sensitive indicator of individual DNA damage repair capacity. In conclusion, we have shown that photon and particle irradiation induce similar transcriptional pathways, albeit with variable amplitude and timing, but also elicit radiation type-specific responses that may have implications for cancer progression and treatment

## Introduction

The use of charged particles is a promising modality in cancer therapy. Particle therapy, which uses focused beams of charged particles such as protons and carbon ions, has become the treatment of choice for targeting specific solid tumors (1, 2), which plays an important role in tumor management particularly in pediatric patients (3). The main advantage of charged particle beams is the possibility to target the tumor more precisely, while the surrounding healthy tissues receive a lower dose as compared to conventional photon radiotherapy (4). This reduces the chance of secondary cancer development (5) and impairment of the immune system (6). However, high linear energy transfer (LET) radiation, like for instance carbon ions, has a higher relative biological effectiveness (RBE) compared to conventional low-LET photon therapy (7), as particles deposit their energy in a more focused manner and therefore result in more complex clustered DNA damage which is more lethal to the tumor cells (8) but may also affect the healthy tissue. Moreover, high-LET radiation is also characterized by higher RBE in terms of other endpoints, such as chromosome aberrations, genetic alterations and normal tissue damage (9). Normal tissue damage is a complex process, which is not solely caused by cell death (10). Radiation-induced DNA damage also triggers changes in chemokine and cytokine production, cell-cell interactions, influx of inflammatory cells and the induction of restorative processes in healthy tissues (11). Genes involved in DNA damage repair, apoptosis, proliferation and inflammatory processes therefore also play a role in the normal tissue response to irradiation (12).

A second important field where charged particles are of relevance is human space exploration. The more feasible and realistic long-term and interplanetary space missions and commercial space flights will become, the more concern they will raise about possible health risks due to exposure to cosmic radiation (13). Astronauts in deep space are subjected to galactic cosmic rays (GCR) and solar particle events (SPE), which result in levels of radiation hundreds of times higher than on Earth. The GCR spectrum is composed of about 87% high energy protons, 12% alpha-particles and 1% of heavier ions up to iron (HZE particles) (14) which are extremely penetrating and difficult to shield (15). Though the HZE particles are less abundant, they possess significantly higher ionizing power with a greater potential for radiation induced damage and, consequently, health effects (16). SPE consist of low to medium energy protons and alpha-particles. Up to now, the assessment of radiation risk for astronauts is almost completely based on extrapolation from epidemiological data on low-LET exposures. Therefore, comprehensive models and radiobiological studies comparing the biological response to different radiation types are needed to validate this approach (17).

The particles and energies which are most often used for particle therapy partially overlap with the lower range of charge and energies (Z=1-26 and approximately 100-1000 MeV/nucleon) of the ions that constitute cosmic radiation. Although the exposure conditions (low *vs*. high doses, low *vs*. high dose rates, whole body *vs*. partial body exposure) and relevant biological endpoints (cancer induction *vs*. tumor cell killing) are different in space and during particle therapy treatment, it is well accepted that a substantial overlap exists in several research topics, e.g. individual radiosensitivity, late stochastic effects such as cancer induction (18, 19) and modulation of the immune system (20). Understanding the cellular radiation response and the processes governing individual sensitivity to high-LET radiation is of pivotal importance in rational choice of radiotherapy treatment schemes. The same holds true for the risk assessment of astronauts and the development of effective protection measures.

Radiobiological transcriptional studies can offer valuable insight in this regard, revealing the biological basis of the cellular response to different radiation types (21, 22). Peripheral blood is an easily-accessible biological sample which allows minimally invasive testing. Furthermore, blood cells are continuously exposed to radiation during radiotherapy and are often used as a surrogate tissue for damage-based radiation biodosimentry and normal tissue radiation sensitivity assessment (23). However, to date, there have been only a limited number of studies comparing gene expression profiles following exposure to low- and high-LET radiation in human peripheral blood cells exposed *in vitro* to α-particles and X-rays (24), neutrons and X-rays (25), mixed-field neutron/X-rays (26, 27), or mouse blood cells exposed *in vivo* to neutrons and X-rays (28).

To add to the knowledge of the cellular response to low- and high-LET radiation, we compared in this study the transcriptional profiles of peripheral blood mononuclear cells (PBMCs) from healthy donors after the cells had been exposed to X-rays, carbon ions or iron ions. We identified specific biological processes that were induced by exposure to heavy ions or X-rays, as well as processes shared by both types of radiation. Our results provide an important basis for further detailed investigation of the differential cellular and tissue response to high- and low-LET radiation.

## Materials and Methods

**Figure 1:**
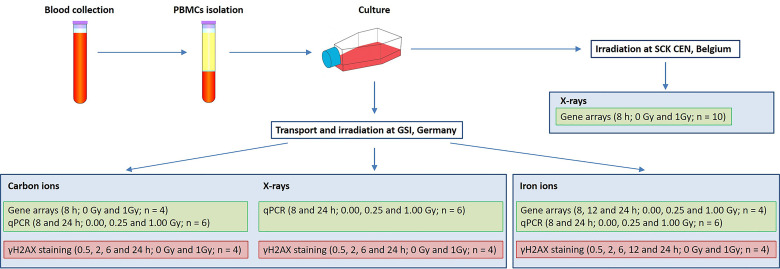
Experimental setup. Samples listed in the same blue box were irradiated in parallel.

In the present study two aspects of biological response to radiation exposure were assessed: gene expression changes (using microarray technology with further qRT-PCR validation) and DNA damage repair (using γH2AX immunofluorescent staining). Irradiations were performed at SCK CEN, Belgium (X-rays) and at GSI Helmholtzzentrum für Schwerionenforschung (abbreviated GSI), Germany (X-rays, iron and carbon ions). Blood samples were collected on three different days, corresponding to three microarray experiments: X-rays (10 donors), carbon (7 donors) and iron ion irradiation (6 donors). The exact number of samples used for every assay is indicated in **Figure 1**, not all the samples could be used for all the assays due to insufficient RNA quality or insufficient number of cells to run all the assays. Matched samples were used for iron ion experiment only. **Figure 1** gives an overview of experimental conditions and number of samples used for every assay. Detailed description of all experimental procedures is given below.

### Blood Collection and PBMCs Isolation

Peripheral blood samples were collected from healthy donors in 9 ml EDTA vacutainer tubes. Blood collection was approved by the local SCK CEN Ethics Committee and was carried out in accordance with the ethical standards of the Helsinki Declaration of 1975, as revised in 2000. Prior to blood donation all the donors involved in the present study signed an informed consent form. Within 30-60 min of blood drawing, PBMCs were isolated by centrifugation on Histopaque-1077 density gradient (Sigma-Aldrich, Bornem, Belgium) according to the manufacturer’s instructions. Isolated cells were suspended at a density of 10^6^ cells/ml in LGM-3 culture medium (Lonza, Walkersville, MD, USA) and were allowed to equilibrate to culture conditions at 37°C in a humidified 5% CO_2_ atmosphere.

### Cell Cultures

Three human lymphoblastoid cell lines TK6, WTK1 and NH32 were used in this study. TK6 cell line was purchased from DSMZ, Leibniz-Institute, Braunschweig, Germany, while WTK1 and NH32 cells were a generous gift from the laboratory of Prof Schwartz, University of Washington. All three cell lines have the same progenitor, WIL2 cell line (29). The TK6 cells express wild-type p53, while WTK1 cells overexpress a mutant form of p53 due to methionine to isoleucine substitution at codon 237 and no wild-type p53 protein (30). The NH32 cell line is genetically homologous to TK6, but its *TP53* gene has been genetically inactivated by a homozygous knockout (31). Cell lines were maintained as exponentially growing stationary cultures in RPMI-1640 medium supplemented with 10% heat-inactivated foetal bovine serum at 37°C in 5% CO_2_ at densities of 4–10 × 10^5^ cells/ml. Cell line irradiations were performed in three replicates (1 × 10^6^ cells per sample) as described below. Following irradiations cells were incubated at standard conditions for 24 h prior to RNA extraction.

### *In Vitro* Irradiation

X-ray, carbon and iron ion irradiations were performed independently, on different days. X-ray irradiation experiments were performed at the irradiation facility at SCK CEN, Belgian Nuclear Research Centre, Mol, Belgium (for microarrays) and at GSI, Darmstadt, Germany (for qRT-PCR validation and γH2AX staining). At SCK CEN, PBMCs were exposed to 1.00 Gy of X-rays, using a Pantak HF420RX machine (250 kV, 15 mA, dose rate of 0.26 Gy/min). Cells were irradiated “free-in-air” at 21°C in a horizontal position with single doses of 0.1 and 1.0 Gy of X-rays from a Pantak HF420 RX generator at an air kerma (K_air_) rate of 0.26 Gy/min or were sham-irradiated. The beam quality can be approximated to H-250 (ISO4037): 250 kV, 15 mA, 1.2 mm Al equivalent inherent filtration and 1 mm Cu additional filtration. First HVL was 2.43 mm Cu and the second HVL was 3.52 mm Cu. The K_air_ at the reference position was measured using a NE2571 ionization chamber (SN309) connected to a Farmer 2500 electrometer. The chamber, together with the electrometer, was calibrated in terms of K_air_ and the traceability to the international standards was assured. The reference point of the ionization chamber was placed at the same distance with the reference position of the samples. The ionization chamber was always placed in the beam, next to the samples, for a precise measurement of the time integrated K_air_. The stability of the X-ray generator during the irradiation was verified in this way using a monitor chamber. Cell line samples were exposed to 1.00 and 3.00 Gy at SCK CEN following the same procedures. For samples that were irradiated at GSI, freshly isolated PBMCs were transported for 4 h by car to GSI using a transportable incubator. X-ray exposures at GSI were performed using an IV320-13 X-ray tube (250 keV, 16 mA, dose rate of 0.5 Gy/min; Seifert, Germany) at 0.25 and 1.00 Gy. Irradiation with heavy ions was performed at GSI on the heavy-ion synchrotron SIS. Carbon ion exposure (0.25 and 1.00 Gy) was performed in the middle of a 25 mm spread-out Bragg peak (center depth 42.5 mm, realized with a PMMA bolus), obtained by active energy variation of the beam in the range of 114.6 – 158.4 MeV/nucleon. Accordingly, the dose averaged LET at the proximal and distal part of the samples (5-ml plastic tube, inside diameter 10 mm) was 60-80 keV/µm. Irradiation with iron ions (0.25 and 1.00 Gy) was performed with a monoenergetic beam (1 GeV/nucleon; LET 155 keV/µm). The beam monitor calibration was performed according to the procedure described by Luoni et al. (32). For verification of the applied dose, additional absolute dosimetry was performed by measuring the absorbed dose to water using a PTW TM30013 Farmer ionization chamber positioned at the sample depth. The dose-averaged LET was calculated using the TRiP98 treatment planning system (33). Sham-irradiated samples were always subjected to the same procedures as the irradiated ones, except for the radiation exposure itself. After *in vitro* irradiation, cells were incubated at 37°C in a humidified 5% CO_2_ atmosphere for the indicated time until further processing.

### RNA Extraction

For RNA isolation, a combination of the TRIzol^®^ reagent (Invitrogen, Carlsbad, CA, USA) extraction method and the clean-up on Qiagen RNeasy columns (Qiagen, Venlo, The Netherlands) was used. Briefly, 5 x 10^6^ cells were lysed in 1 ml of TRIzol^®^ reagent and further processed following the manufacturer’s recommendations. Following the RNA precipitation with isopropanol, the obtained pellet was re-suspended in 1 ml of ethanol and transferred to the RNeasy column. Further purification was done according to the manufacturer’s instructions. RNA concentration was measured on a NanoDrop-2000 spectrophotometer (Thermo Scientific, Erembodegem, Belgium) and the quality of total RNA samples was assessed using Agilent 2100 Bioanalyzer (Agilent Technologies, Santa Clara, CA, USA). Only samples with an RNA integrity number >7 were considered as suitable for further microarray hybridization. RNA extraction from cell line samples was performed following the same procedures.

### Microarray Hybridization

Gene expression profiling was performed using the GeneChip^®^ Human Gene 1.0 ST Array (Affymetrix, Santa Clara, CA, USA) which interrogates 28,536 well-annotated genes with 253,002 distinct probe sets, allowing expression analysis at both gene and exon level. Ten µg of cRNA, synthesized and purified from 0.25 µg of total RNA using the Ambion^®^ WT Expression kit (Ambion, USA) was used for cDNA synthesis, followed by cDNA fragmentation and labeling with the GeneChip^®^ Terminal Labeling kit (Affymetrix). Fragmented and labeled cDNA was hybridized to Human Gene 1.0 ST arrays (Affymetrix) using the GeneChip^®^ Hybridization, Wash and Stain kit (Affymetrix) (hybridization module) and hybridization controls (Affymetrix) with rotation at 45°C for 16 hours. After hybridization, arrays were washed and stained using the GeneChip^®^ Hybridization, Wash and Stain kit (stain module) after which the arrays were immediately scanned using an Affymetrix GeneChip^®^ Scanner. Raw data of X-ray and heavy ion experiments have been submitted to the ArrayExpress database under accession numbers E-MTAB-3463 and E-MTAB-5761, respectively.

### Microarray Data Analysis

The obtained microarray data were imported into Partek Genomics Suite, version 6.6 (Partek Inc., St Louis, MO, USA) as.CEL-files. The probe summarization and probe set normalization were done using the Robust Multichip Analysis (RMA) algorithm (34) which includes background correction, quantile normalization and log_2_ transformation. Microarray data were analyzed using ANOVA with dose, donor and time point (whenever applicable) as factors. To correct for multiple testing, we used the false discovery rate (FDR) as described by Benjamini and Hochberg (35) to adjust *p*-values (FDR < 0.05). Genes were considered significantly differentially expressed between the two groups if adjusted *p*-values were < 0.05. In some cases, a more stringent additional cut-off of fold-change ≥|2| was used, as explained in the text.

We also performed Alternative Splicing ANOVA in Partek to detect genes which were alternatively spliced in response to different radiation types. An FDR-corrected *p*-value of < 0.05 was considered significant for alternative splicing events. To further reduce the number of false positives, the probe sets with log_2_ values below the noise level in all samples were excluded from analysis, except for the cases where there was a significant difference in expression of a single exon between the groups (*p* < 0.05).

The Venny on-line tool (36) was used to compare gene lists and create Venn diagrams: http://bioinfogp.cnb.csic.es/tools/venny/index.html.

### Reverse Transcription and qRT-PCR

The following genes were selected for qRT-PCR validation: *PCNA, GADD45A, RPS27L, ASTN2, NDUFAF6, FDXR, MAMDC4*. The same RNA samples as those used for microarray hybridization plus two additional samples irradiated on the same day but not selected for microarray hybridization (n=6), were used for cDNA synthesis with the GoScript™ Reverse Transcription System (Promega, Leiden, The Netherlands) with random hexamer primers. For each gene, qRT-PCR reactions were run in duplicate using the MESA GREEN^®^ qRT-PCR kit (Eurogentec, Seraing, Belgium) on an Applied Biosystems^®^ 7500 Real-Time PCR instrument following the manufacturer’s instructions. To determine the efficiency and specificity of the designed primers, a standard curve experiment with melt curve was run for every primer pair. qRT-PCR data were analyzed by 7500 Software v2.0.6 and Microsoft Excel using the Pfaffl method (37). The relative amount of transcript of the selected genes was normalized to *PGK1* and *HPRT1* using the geometric mean of these reference genes (38). cDNA synthesis and qRT-PCR of the cell line samples was performed following identical procedures for the following genes: *EDA2R*, *NDUFAF6*, *PTPN14* and *VWCE*. All primer sequences can be found in **Supplementary Table 1**.

### Rank-Rank Hypergeometric Overlap (RRHO) Analysis

The RRHO algorithm allows for the comparison of two microarray datasets. Each dataset is processed as a ranked list based on expression differences between two classes of samples (0 Gy and 1 Gy, in our case). RRHO analysis (39) was performed using the online tool (http://systems.crump.ucla.edu/rankrank/index.php). As this algorithm only allows the comparison of two gene lists at a time, the following comparisons were performed: X-rays *vs* carbon ions, X-rays *vs* iron ions and carbon ions *vs* iron ions using a step size of 100.

### Transcription Factor and Gene Ontology Terms Enrichment Analysis

Transcription factor and Gene Ontology terms enrichment analysis was performed using the Enrichr online tool (http://amp.pharm.mssm.edu/Enrichr/) (40, 41) which uses input gene lists to calculate enrichment of genes based on different databases of chromatin immunoprecipitation experiments and Ontologies. We used the “ENCODE and ChEA Consensus TFs from ChIP-X” and “GO Biological Process 2015” databases to calculate enrichment of transcription factor binding and biological processes, respectively, in significantly differentially expressed genes. The same tool was used to calculate enrichment of GO biological processes terms in significantly alternatively spliced genes.

### Gene Set Enrichment Analysis (GSEA)

Gene set enrichment analysis (42) was performed using default settings: the significance of the normalized enrichment score for each gene set was assessed through 1000 gene set permutations. Gene sets with an FDR q-value < 0.25 were considered significant, as suggested by the GSEA tutorial. For each radiation type, 1-Gy and sham-irradiated samples analyzed at 8 h after exposure were used for comparison. To have a general view of response to each radiation type, Hallmark Gene Sets collection of the Molecular Signatures Database was used. This collection consists of 50 gene sets representing specific well-defined biological states and processes, which helps to reduce noise and redundancy in different available databases and provides a better delineated biological space for GSEA.

### γH2AX Foci Detection Using Fluorescent Microscopy

PBMCs from four donors were fixed in 4% paraformaldehyde (Merck KGaA, Darmstadt, Germany) at several time points (see **Figure 1**). Following the fixation step, cells were cytospun on glass slides using Shandon™ EZ Double Cytofunnels™ and permeabilized with 0.25% Triton X 100 (Sigma Aldrich, Belgium) for 5 min, blocked with 3% bovine serum albumin (Sigma Aldrich, Belgium) for 30 min and incubated overnight at room temperature with monoclonal mouse anti-γH2AX (phospho S139) antibody [3F2] (ab22551, Abcam, Cambridge, MA, USA) at 4°C. Cells were then incubated for 1 h with polyclonal goat anti-mouse secondary antibody coupled to FITC (F2012, Sigma Aldrich, Belgium) at 37°C and then mounted in Vectashield mounting medium containing DAPI (Vector Laboratories, Burlingame, CA, USA). Between each of the previous steps, the slides were washed with phosphate-buffered saline.

An automated inverted fluorescence microscope (Eclipse Ti, Nikon, Tokyo, Japan), equipped with a motorized XYZ stage was used for image acquisition of immunostained slides. Images were acquired with a 40X Plan Fluor oil objective (Numerical aperture 1.3) and an Andor iXon3 camera (Andor Technology, South Windsor, CT, USA), providing images with a lateral resolution of 0.2 µm/pixel. For each sample, 25 fields were acquired on 7 Z-planes (separated by 1 μm). The obtained images were analyzed with the CellBlocks.ijm script (43), written for FIJI image analysis freeware (44), essentially as described before (45). In brief, the image analysis workflow starts by segmenting each nucleus in the DAPI channel, using an automatic thresholding algorithm, after noise reduction and flat field correction. Subsequently, γH2AX foci signals are selectively enhanced by means of a multiscale Laplacian and segmented by means of automatic thresholding. Within each nucleus, the intensity of the γH2AX channel is measured along with the number of γH2AX foci and the foci occupancy, *i.e.*, the total projected area of the nucleus that is occupied by spots (total spot area divided by the nucleus area). On average, 500 nuclei were analyzed per sample.

## Results

### Gene Level Transcriptome Analysis Shows a Common p53-Regulated Gene Expression Signature After Low- and High-LET Irradiation

**Figure 2:**
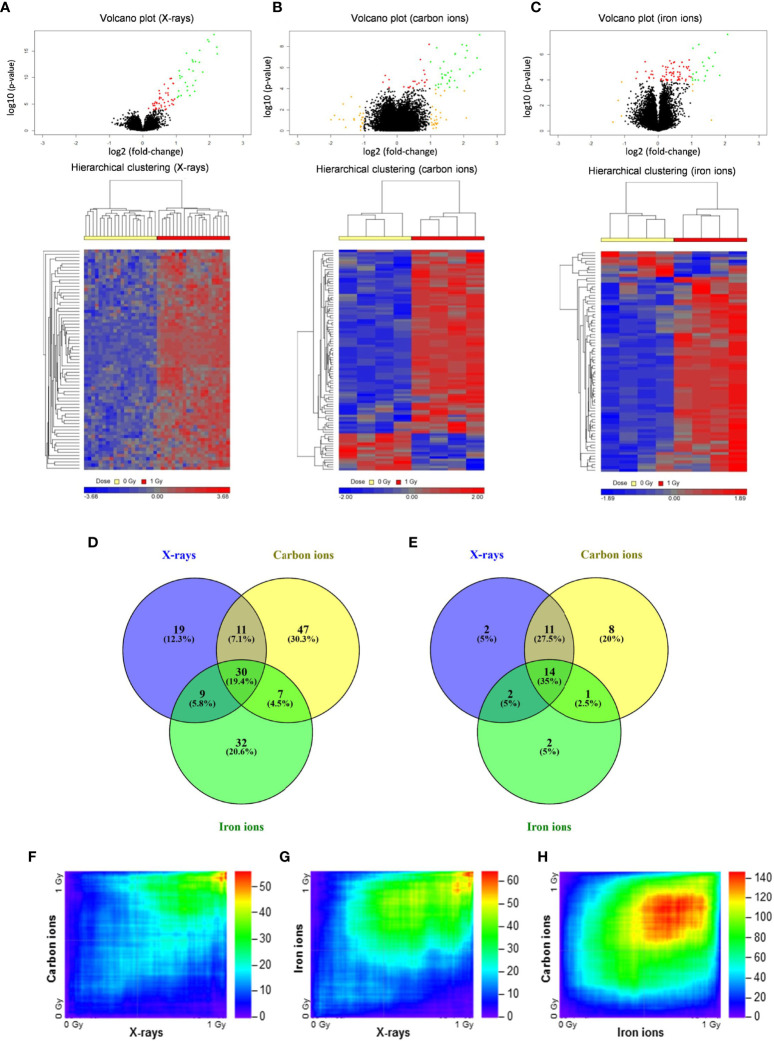
Changes in gene expression in PBMCs after exposure to X-rays, carbon ions and iron ions. **(A–C)** Volcano plots and heatmaps of gene expression changes between controls and cells irradiated with X-rays **(A)**, carbon ions **(B)** or iron ions **(C)** at 8 h after exposure. Red points on volcano plots indicate genes with FDR <0.05, orange points indicate genes with |FC| >2 and green points indicate genes with FDR <0.05 and |FC| >2. Heatmaps show expression profiles of differentially expressed genes with an FDR <0.05. **(D, E)** Venn diagrams showing overlap in differentially expressed genes with FDR <0.05 **(D)** or FDR <0.05 and |FC| >2 **(E)** between the different radiation types. **(F–H)** Rank-rank hypergeometric overlap heatmaps indicating overlap in gene expression changes between X-rays and carbon ions **(F)**, between X-rays and iron ions **(G)**, and between carbon ions and iron ions **(H)**. Color scale bars indicate the log_10_-transformed hypergeometric *p*-values.

To compare the effects of high- and low-LET radiation exposure on gene expression in human PBMCs, microarray analysis was performed at 8 h after exposure to 1 Gy of X-rays, carbon ions or iron ions. This time point was chosen because we observed a prominent gene expression response after 8 h in our previous studies (46, 47), moreover we found that prolonged culturing times trigger activation of apoptosis-related genes also in control samples, which might complicate data interpretation (48). We also observed dose-dependent gene expression up-regulation for doses ranging from 0.025 to 2.00 Gy, however, following 2.00 Gy exposure a clear saturation of the effect was observed (48), therefore in this study we opted for using 1 Gy for all PBMCs exposures. Following the exposure to X-rays, carbon ions or iron ions, 69, 95 and 78 genes, respectively, were detected as differentially expressed (FDR-corrected *p* < 0.05) compared to control samples (**Figures 2A**–**E** and **Supplementary Tables 2**–**4**). The majority of these genes were induced after irradiation (**Figures 2A**–**E**), including 30 genes that were differentially expressed in response to all radiation types. Of these, 14 genes were up-regulated more than 2-fold (**Figure 2E**). The lists of genes differentially expressed exclusively following the exposure to X-rays, carbon or iron ions can be found in **Supplementary Tables 2**–**4**, respectively.

When comparing two independent high-throughput gene expression experiments with different sample numbers, threshold-free methods outperform threshold-based ones in providing reliable results (39). Thus, to obtain a better impression of the similarity in gene expression after exposure to different radiation types, the Rank-rank Hypergeometric Overlap algorithm was used. This revealed a very significant degree of overlap among the genes up-regulated in both conditions (shown in the top right corner of the heatmap) for the comparisons between X-rays and carbon ions (47 overlapping genes, lowest p≈10^-56^) (**Figure 2F**) as well as X-rays and iron ions (59 overlapping genes, lowest p≈10^-65^) (**Figure 2G**). However, for the comparison between the two high-LET ions (**Figure 2H**) the degree of overlap was much more significant (1715 overlapping genes, lowest p≈10^-146^). Together, our data show that irrespective of the radiation type, the majority of the affected genes are up-regulated after exposure, and that the identity of these genes is highly similar, although some radiation type-specific genes do seem to exist.

**Figure 3:**
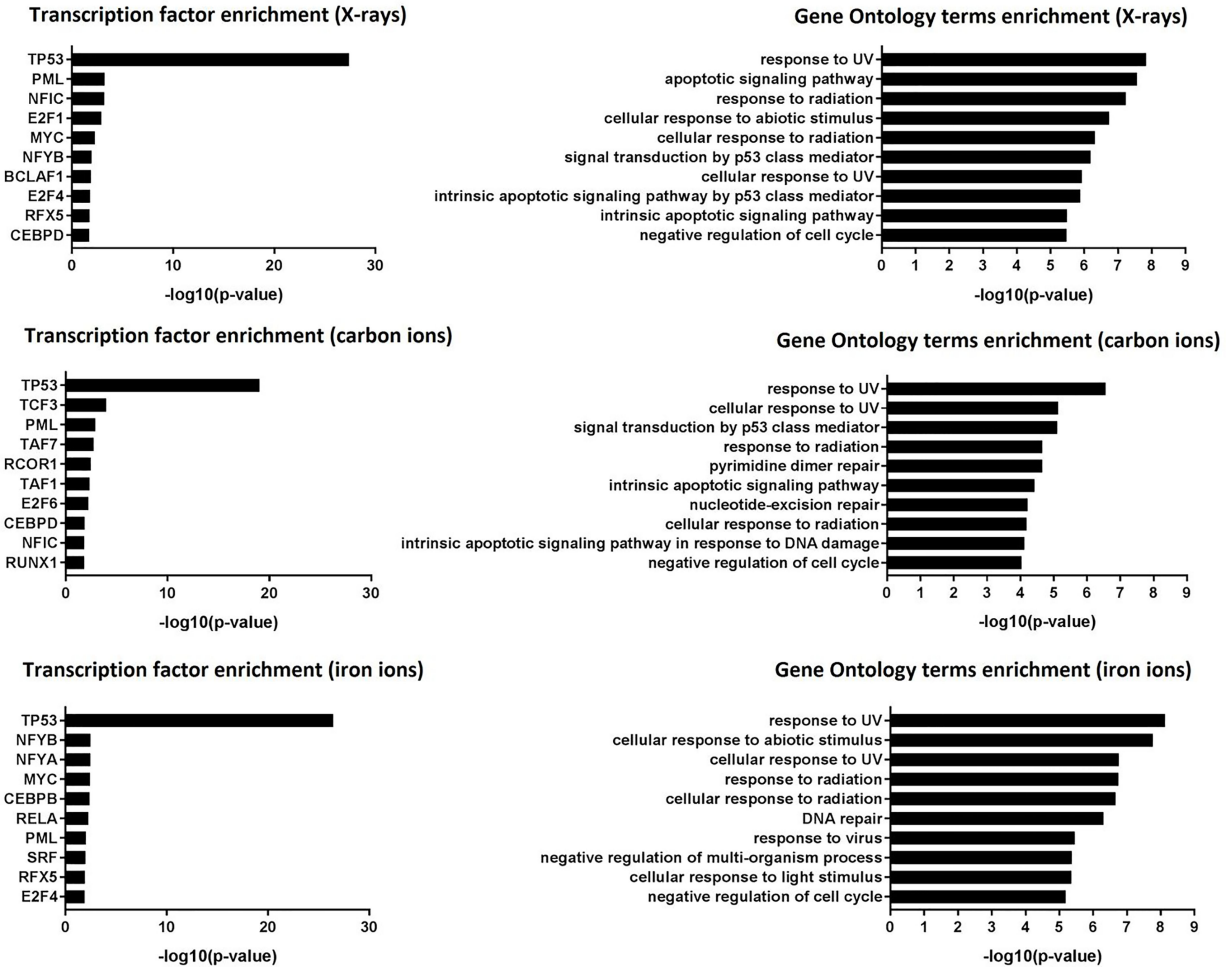
Transcription factor enrichment and GO term enrichment. Left panel: transcription factor enrichment results following exposure to X-rays, carbon ions or iron ions. Right panel: Biological processes that are mostly affected following exposure to X-rays, carbon ions or iron ions, based on gene level analysis.

Transcription factor enrichment analysis suggested that, for all radiation types, the affected genes were transcriptionally regulated by p53 (**Figure 3**, left panel), and they were enriched in functions related to canonical p53-dependent pathways such as response to (ultra violet) radiation, negative regulation of the cell cycle, DNA repair and apoptosis (**Figure 3**, right panel).

The list of p53 targets is constantly growing and it remains probably the most studied transcription factor. There are at least 350 confirmed p53 targets and over 3500 potential targets (49). To confirm the p53-dependent activation of some of the significantly up-regulated genes, three lymphoblastoid cell lines with different p53 status were irradiated with different doses of X-rays (0 Gy, 1 Gy and 3 Gy). qRT-PCR was performed to measure gene expression of selected hits *EDA2R*, *NDUFAF6*, *PTPN14* and *VWCE*, all of which were induced after X-ray, carbon and iron irradiation. *EDA2R* is a well-known *bona fide* p53 target (50), while PTPN14 has been recently identified as such (51). *VWCE* is often found in genome-wide data sets of activated p53 targets (49). *NDUFAF6* has not yet been validated as a direct p53 target gene, but was identified to be radiation-responsive in our previous study (47). In TK6 cells, which have wild-type p53, we observed a significant, dose-dependent induction of all four genes. In contrast, none of these genes were induced after irradiation of WTK1 (p53 mutated) or NH32 (p53 null) cells (**Figure S1**).

### Gene Set Enrichment Analysis (GSEA) Reveals Stronger Enrichment of Immune Response and Inflammation-Related Gene Sets by High-LET Radiation

**Figure 4:**
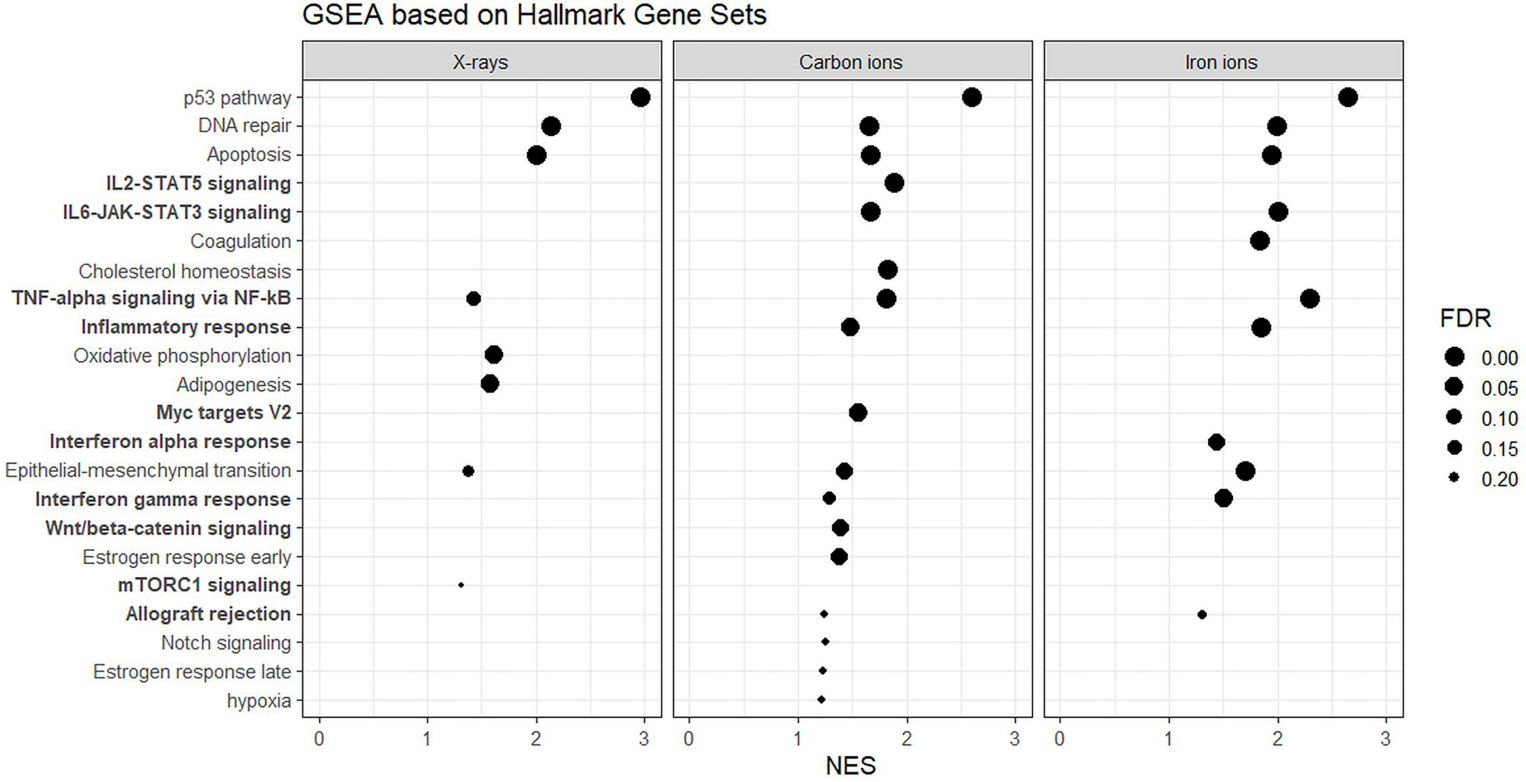
GSEA based on Hallmark Gene Sets. Gene sets related to the immune system and inflammation are in bold. NES, normalized enrichment score; FDR, false discovery rate q-value.

**Figure 5:**
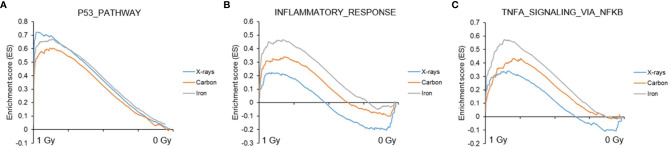
GSEA analysis. GSEA enrichment plots for three gene sets following exposure to X-rays, carbon or iron ions: **(A)** p53 pathway, **(B)** Inflammatory response, **(C)** TNFα signaling via NF-κB. Gene sets with a distinct peak at the beginning or the end of the ranked list are generally the most relevant, indicating that this specific gene set is enriched in up- or down-regulated genes, respectively.

Next, to detect modest but coordinated changes, we performed GSEA (52). A classical DNA damage response, with p53-pathway, apoptosis and DNA damage repair-related gene sets being very significantly enriched in up-regulated genes was observed after exposure to 1 Gy of all radiation types (**Figures 4** and **5A**). Interestingly, especially after exposure to heavy ions, also several immune response and inflammation-related gene sets were identified as significantly enriched in irradiated samples (**Figures 4** and **5B, C**). For instance, genes related to the inflammatory response showed no preferential enrichment in either sham- or X-irradiated PBMCs. In contrast, exposure to heavy ions, especially iron ions, resulted in a significant up-regulation of these genes (**Figure 5B**). Similarly, the radiation effect on genes involved in TNFα signaling *via* NF-κB was more pronounced after heavy ion irradiation as compared to X-irradiation (**Figure 5C**). Together, these results corroborate the observation that exposure of PBMCs to heavy ion irradiation induces similar pathways but with more pronounced changes in gene expression when compared to X-rays, while certain pathways, especially those related to inflammation are particularly triggered by heavy ions.

### qRT-PCR Analysis Shows Radiation Type- and Time-Dependent Gene Expression Response

**Figure 6:**
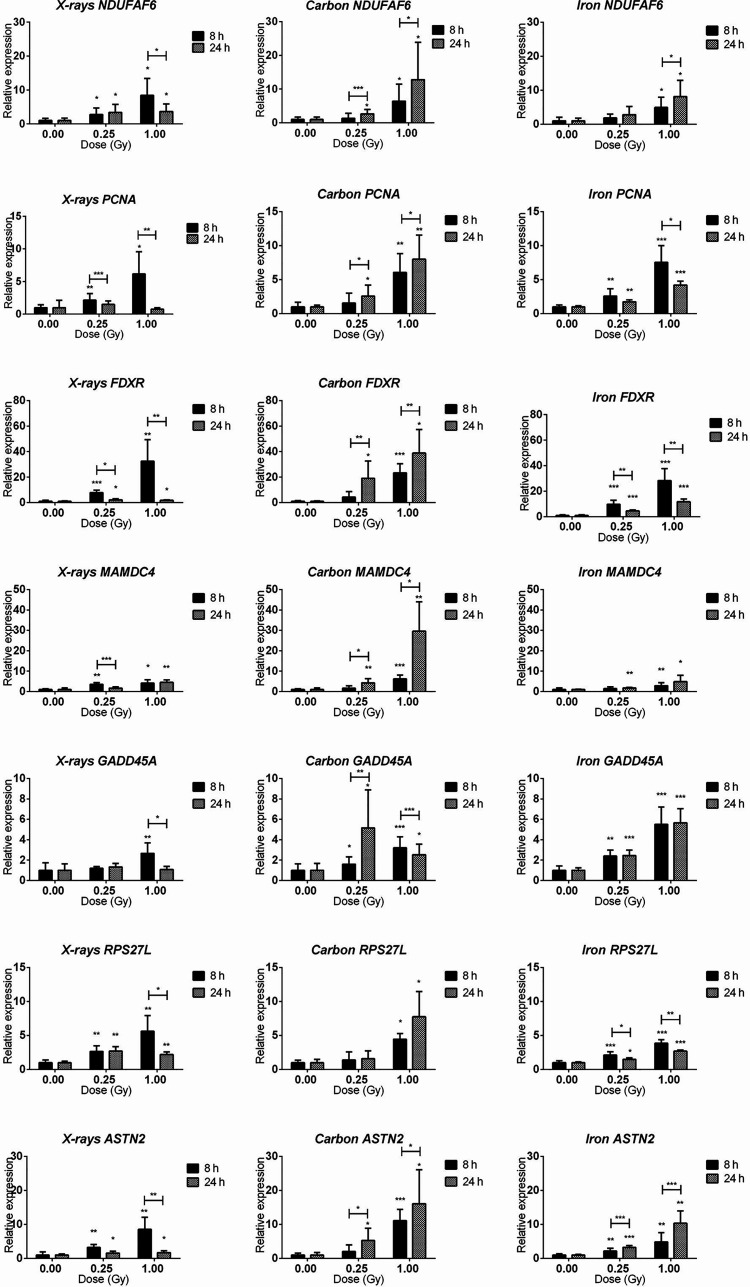
qRT-PCR validation of the microarray results. qRT-PCR results for *NDUFAF6, PCNA, FDXR, MAMDC4, GADD45A, RPS27L*, and *ASTN2* genes (shown in rows) at 8 and 24 h after irradiation with 0.25 and 1.00 Gy of X-rays, carbon or iron ions (shown in columns). Graphs represent mean of six biological replicates + standard deviation. Statistical comparison between samples irradiated with different doses at two different time points was performed using 2-way ANOVA with Bonferroni *post-hoc* test (*p < 0.05, **p < 0.005, ***p < 0.0001).

Seven genes (*PCNA*, *GADD45A*, *RPS27L*, *ASTN2*, *NDUFAF6*, *FDXR*, *MAMDC4)* were selected for qRT-PCR validation of microarray data and temporal follow-up. The rationale of gene selection for qRT-PCR validation is explained below. *PCNA*, *FDXR*, *GADD45A* and *RPS27L* were significantly up-regulated 8 h after exposure to all radiation types, while *ASTN2* showed significant up-regulation after exposure to X-rays and iron ions, but not to carbon ions according to the microarray data. *NDUFAF6* and *MAMDC4* were alternatively spliced (see next section for detailed description) in response to exposure to all radiation types. To obtain better insight in the dose- and time-dependence of expression of these genes, a lower dose (0.25 Gy) as well as an additional time point (24 h) were included (**Figure 6**). All selected genes showed significant up-regulation at 8 h after exposure to 1 Gy of all radiation types. In many cases their induction was significant even at a lower dose of 0.25 Gy. *FDXR* showed the highest degree of induction following the exposure to all radiation types. The expression of *ASTN2*, in contrast to microarray results, was also significantly induced by carbon ions. Importantly, while all genes, except *MAMDC4*, reduced in expression with time in X-irradiated cells, their up-regulation was in general retained, or often even further induced in cells exposed to heavy ions, especially in the case of carbon ions.

### Transcript Variation Is Induced by Low- and High-LET Radiation

**Figure 7:**
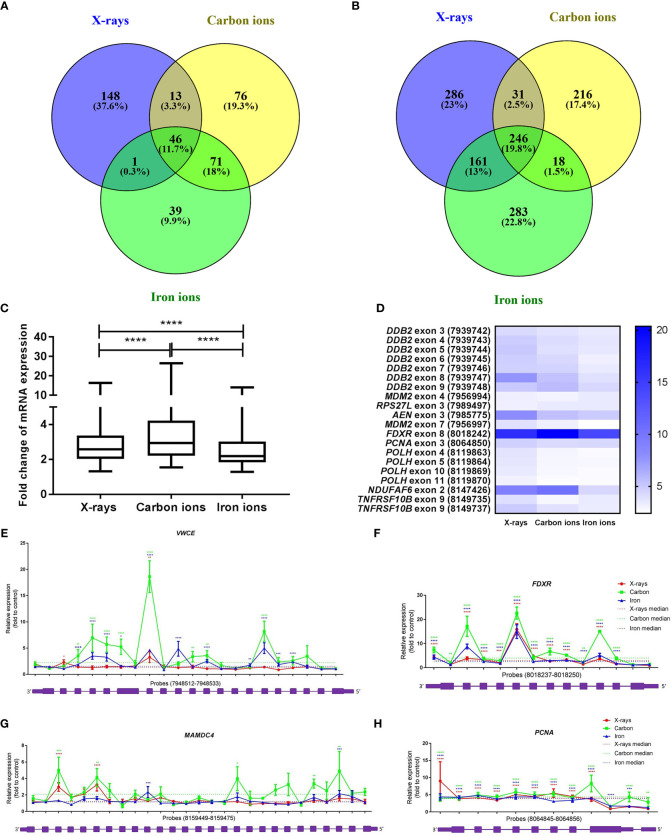
Radiation-induced alternative splicing. **(A)** Venn diagram showing the number of alternatively spliced genes with FDR-corrected p-value < 0.05 at 8 h after exposure to 1 Gy of X-rays, carbon or iron ions**. (B)** Venn diagram showing the number of differentially expressed exons with FDR-corrected p-value < 0.05 at 8 h after exposure to 1 Gy of X-rays, carbon or iron ions. **(C)** Changes in exon expression induced at 8 hours after exposure to 1 Gy of different radiation types. Centerlines show the median, boxes represent the range between the first and third quartiles and whiskers represent the highest and lowest values. Statistical comparison was performed using Wilcoxon matched-pairs signed rank test (****p-value < 0.0001). **(D)** Heatmap showing fold-changes in the expression of the 20-exon signature (probe set numbers are shown in brackets) 8 hours after exposure to 1 Gy of different radiation types. This 20-exon signature was identified as particularly responsive to X-ray exposure (47). **(E–H)** Alternative transcription/splicing of *VWCE*, *FDXR*, *MAMDC4* and *PCNA* genes at 8 h after exposure to 1 Gy of X-rays, carbon or iron ions. Genomic organization of each gene is shown below the graph in purple; every box represents an exon of the gene, schematic representation of the exons does not correspond to their actual size. Fold-changes to control values are shown for every probe set specific to each exon of the gene. Median fold-change to control value for each radiation type is shown with the dotted line. Error bars represent SEM (n = 10 for X-rays, n = 4 for carbon and iron ions). Statistical comparison between irradiated and non-irradiated samples was performed using repeated measures 2-way ANOVA with Sidak’s *post-hoc* test (*p < 0.05, **p < 0.005, ***p < 0.001, ****p < 0.0001).

Exposure to low-LET radiation not only changes gene expression but also triggers the production of alternative transcripts (due to alternative splicing or transcription) (47, 53–55). A core signature of genes that become alternatively spliced in response to all radiation types was identified (**Figure 7A**). The majority of these genes were also differentially expressed at the gene level (36 out of 46), which aligned well with our previous results (47). More overlap was observed between the iron- and carbon-ion irradiated groups – 33% of the genes were in common, while between the X-ray irradiated cells and heavy-ion irradiated cells the overlap was only about 15%. We also compared the number of differentially expressed exons between different radiation types to assess the levels of induction of transcript variants. Exposure to 1 Gy of X-rays resulted in significant (FDR < 0.05) up-regulation of 724 exons, to carbon ions – of 511 exons and to iron ions – of 708 exons (**Supplementary Table 6**). In this case, more overlap was observed between iron ions and X-rays – 32.8% of exons were in common (**Figure 7B**). When comparing the fold-changes in expression of the overlapping 246 exons (**Supplementary Table 6**), the highest induction levels were shown by carbon ions (**Figure 7C**). In addition, changes in expression of the 20-exon signature identified earlier (47) were compared between different radiation types (**Supplementary Table 6**). These 20 exons are particularly responsive to X-rays and important for the sample classification according to the exposure dose. The comparison revealed that most of the above-mentioned 20 exons are in general less responsive to heavy ions compared to X-irradiation (**Figure 7D**). The detailed results for four genes overlapping for all radiation types (*PCNA*, *VWCE*, *FDXR* and *MAMDC4*) are shown in **Figures 7E**–**H**. In this case, the most pronounced alternative splicing response was observed after carbon ions exposure. This was especially the case for *MAMDC4* and *VWCE*. The Gene Ontology Biological Processes terms enriched in alternatively spliced genes common for all radiation types were predominantly related to apoptosis and DNA damage repair (**Supplementary Table 5**). Carbon ion exposure resulted in alternative splicing of several genes coding for classical HLA class I molecules (HLA-A, HLA-B, and HLA-H) and class II molecules (HLA-DMB) and histone-coding genes (HIST2H3A, HIST2H3PS2, HIST2H3C, and HIST2H3D), which was not observed following the exposure to iron ions or X-rays.

### Heavy Ion Exposure Results in Clustered DNA Damage and Slower DNA Damage Repair as Compared to X-Rays

**Figure 8:**
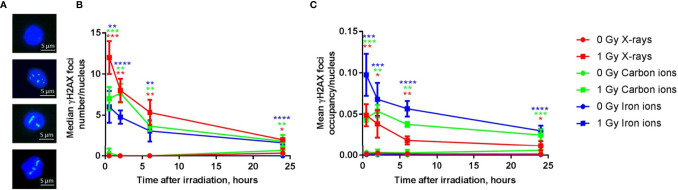
DNA repair kinetics after exposure to different types of radiation. **(A)** Representative examples of immunostained γH2AX foci in PBMCs 6 h following (from top to bottom) sham-irradiation, exposure to 1 Gy of X-rays, iron ions or carbon ions. **(B)** The number of γH2AX foci per nucleus after exposure to 1 Gy of X-rays, carbon and iron ions at different time points (median of three biological replicates for X-rays and carbon ions and four biological replicates for iron ions, error bars represent standard deviations). **(C)** The occupancy of γH2AX foci per nucleus (average of three biological replicates for X-rays and carbon ions and four biological replicates for iron ions, error bars represent standard deviations) after exposure to 1 Gy of X-rays, carbon and iron ions at different time points. Statistical comparison between irradiated and sham-irradiated samples was performed using unpaired *t*-test (*p < 0.05, **p < 0.005, ***p < 0.001, ****p < 0.0001).

Next to a transcriptional profiling, we evaluated the genotoxic impact of radiation exposure in the PBMCs over time for all radiation types. While quantification of DNA damage is commonly performed by counting the number of nuclear γH2AX foci (56, 57), high-LET radiation induces strongly clustered breaks along the track of the beam that may result in few microscopic foci, but with large relative size when a cell is visualized perpendicular to the orientation of the beam track (**Figure 8A**). γH2AX foci nuclear occupancy may therefore be a better measure for damage severity (45). Indeed, when calculated as the number of foci per nucleus, the absolute number of unrepaired breaks after 24 h was similar for all radiation types (**Figure 8B**). However, when considering foci occupancy, the amount of unrepaired DNA double-strand breaks (DSBs) 24 h after irradiation was 23% for X-rays, 42% for carbon ions and 31% for iron ions. When considering the foci occupancy per nucleus, the amount of damage still present 24 h after exposure to iron ions was comparable to the amount of damage observed in X-irradiated cells at 0.5 h (**Figure 8C**). For X-rays and iron ions the maximal foci occupancy was detected at 0.5 h post-irradiation, while for carbon ions this peak was observed at 2 h post-irradiation. The severity of DNA damage and kinetics of the repair were therefore clearly LET-dependent.

### Gene Expression May Serve as a Proxy for DNA Damage Repair Efficiency

**Figure 9:**
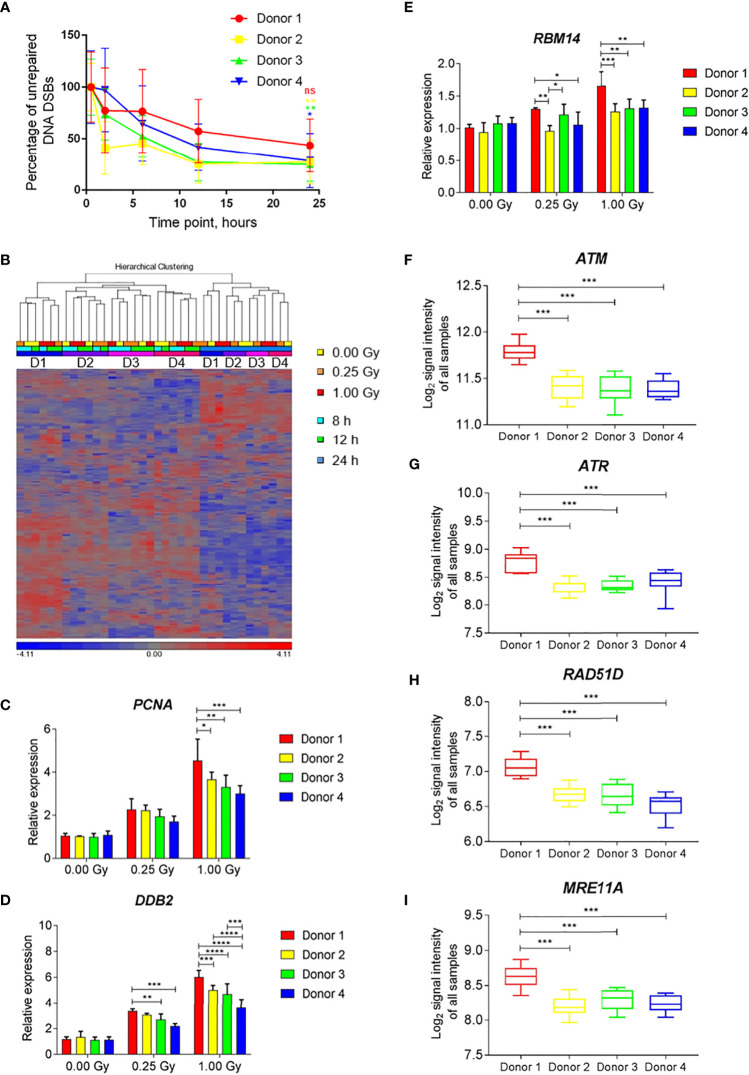
Individual differences in DNA damage repair kinetics and gene expression induced by exposure to iron ions. **(A)** Individual DNA repair kinetics of four donors shown as percentage of gH2AX foci occupancy compared to 1 Gy-irradiated sample at 0.5 h time point (baseline damage subtracted). Error bars represent SEM of 2 technical replicates. Statistical comparison between the amount of damage per donor detected at 0.5 h and 24 h following irradiation was performed using repeated measures 2-way ANOVA with Sidak’s post hoc test (*p < 0.05, **p < 0.005, ns-not significant). **(B)** Hierarchical clustering of DNA repair genes (MsigDB gene set DNA Repair) shows time- and subject-dependent expression. **(C–E)** Dose-dependent expression of selected DNA repair genes shows higher induction in Donor 1 compared to other donors. Bars show the mean of three time points, error bars show SD. Statistical comparison was performed using repeated measures 2-way ANOVA with Tukey’s post-hoc test (*p < 0.05, **p < 0.005, ***p < 0.001, ****p < 0.0001). **(F–I)** Expression levels of selected DNA repair genes shows overall higher expression in Donor 1 compared to other donors. Box plots show the mean of all samples (all doses and time points), whiskers show minimal and maximal values. Statistical comparison was performed using unpaired t-test (***p < 0.001) ns - not significant.

To compare the changes in gene expression with DNA repair kinetics at the level of individual donors, samples of four individuals that were irradiated with iron ions were used. All four individuals showed a clear reduction in the number of DSBs with time (**Figure 9A**). However, for Donor 1 the percentage of unrepaired DNA DSBs after 24 h was 43.6% and the difference between the damage detected at 0.5 h and 24 h time point was not statistically significant. In contrast, for the other donors this difference was significant, and the percentages of unrepaired damage were lower, ranging between 25.2-28.7%.

Hierarchical clustering of the gene expression profiles of DNA repair-related genes showed time- and subject-dependent expression. This resulted in two major clusters of samples depending on the time point, with 24-h samples segregating from 8-h and 12-h samples (**Figure 9B**). Within each time cluster, expression profiles of Donor 1 clustered separately from those of the other three subjects (**Figure 9B**). Several radiation-induced DNA repair genes (e.g., *PCNA*, *DDB2*, *RBM14*) showed an enhanced radiation response in Donor 1 compared to other donors, especially after a high dose (**Figures 9C**–**E**). This donor also showed elevated levels of expression of several DNA damage response-related genes, (e.g., *ATM, ATR, RAD51D, MRE11A*) independent of the irradiation dose and time point (**Figures 9F**–**I**). This indicates that individual differences in the overall and radiation-induced expression levels of DNA repair genes exist, which may possibly explain individual differences in DNA repair kinetics.

## Discussion

In the present study, we compared the genome-wide transcriptional response of human PBMCs after acute exposure to three radiation types with different LET characteristics: X-rays, carbon and iron ions. An equal dose of 1 Gy was used, as our main goal was to identify the differences in response caused by high- and low-LET radiation rather than to compare RBE-weighted doses. It was also previously suggested to compare equal rather than equitoxic doses of high- and low-LET radiation in the context of gene expression analysis (58). In addition, we analyzed the DNA repair kinetics after exposure to the above-mentioned radiation types.

In our study, we found a very similar primary p53-dependent response to all radiation types at 8 h after exposure. The identity of differentially expressed genes was in good accordance with other transcriptional radiobiological studies performed on human blood samples (59). A similar result was obtained by Sokolov and co-authors who showed that gene expression profiles in normal human fibroblasts following γ-radiation and decays of high-LET-like ^125^I share the majority of genes, indicating activation of similar pathways (60). A study by Kurpinski and co-authors showed that most of the differentially expressed genes which were in common after exposure to 1 Gy of X-rays and iron ions in human mesenchymal stem cells were involved in cell cycle and DNA damage response and repair, which is in accordance with our observations (61). Study by Ding et al. on human bronchial epithelial cells exposed to 0.5 and 1 Gy of γ-rays, 1000 MeV/nucleon iron and silicon ions (LET of 150 and 44 keV/µm respectively) showed induction of common gene sets related to cell death, cell cycle regulation, DNA repair and cellular growth and proliferation as well as activation of several p53-regulated genes for all radiation types (62). However, the expression profiles were LET-dependent and distinct enough to classify the samples according to radiation type with very high accuracy (62).

Even though we also found several genes “unique” to a specific radiation type, it is likely that many of them would also respond to the other radiation types in a different experimental set-up (i.e. time-dose combination) due to the differences in RBE and\or gene expression kinetics, but what is already clear from our and other studies is that the magnitude of gene expression changes and the number of differentially expressed genes are consistently higher for high-LET particles (22). Some of the observed differences may be explained by the different nature of X-rays (photons) and heavy ions (particles). The DNA damage caused by particles is more complex and difficult to repair compared to X-rays, as confirmed by slower DNA repair kinetics shown in our study, which may result in different signaling responses. A similar observation was made after exposure to accelerated nickel ions, which induced a persistent DNA damage response in endothelial cells up to 24 h after treatment (63). Even though the LET of iron ions was higher than that of carbon ions we observed a more pronounced response after exposure to the latter, for example, in case of gene expression induction, alternative splicing and slower DNA repair kinetics. This observation might possibly be explained by the higher fluence used for the carbon irradiation resulting in cells being hit by more ions, thus other factors and not just the LET value alone should be considered when interpreting the results.

Interestingly, GSEA identified several immune response-related gene sets as significantly enriched specifically in samples irradiated with heavy ions. Paradoxically, radiation was reported to modulate immune responses in a complex dose-dependent manner with possible pro- and anti-inflammatory responses (64). NF-kB is the key transcription factor which plays a central role in regulation of the expression of pro-inflammatory cytokines and chemokines such as TNF-α, IL-1, IL-2, IL-6 and MCP-1 (20, 65). Low-dose radiation is well known to treat benign inflammatory or hypoproliferative conditions (66), and this is thought to be due to inhibition of NF-κB at doses below 2 Gy (67), however, different cell types show different sensitivity toward NF-κB inhibition/activation by ionizing radiation. The possible mechanisms responsible for NF-κB inhibition are the decrease in p38 (an up-stream molecule of NF-κB) (68) and decrease in 26S proteasome activity resulting in prevention of the IκB regulatory complex degradation leading to reduced translocation of NF-κB complex to the nucleus (69). Radiation doses above 2 Gy have conversely been demonstrated to increase the activity of NF-κB (64). In a study by Baumstark-Khan and co-authors high-LET argon ions (272 keV/µm, 95 MeV/nucleon) induced a stronger NF-kB-dependent reporter gene expression compared to X-rays (70). A later study from the same group showed that carbon ions (33 and 73 keV/µm) and X-rays activate NF-kB-dependent gene expression in HEK293 cells 4 h after exposure. However, activation by carbon ions was induced by 1.3 Gy while activation by X-rays required a higher dose of 16 Gy (71). A further group reported NF-κB activation in normal human monocytes exposed to 0.7 Gy of iron ions (72). Ding and co-authors found more significant expression changes in the pro-inflammatory acute phase response pathway in bronchial epithelial cells following the exposure to iron and silicon ions compared to low-LET γ-rays when using equal doses of 0.5 and 1 Gy, which is in accordance with our results (62). We hypothesize that the activation of immune response and inflammation-related gene sets particularly by heavy ions observed in our study is mainly due to the use of equal doses of different radiation types. This observation suggesting overall increase of carcinogenic potential related to NF-kB activation (73, 74) by heavy ions at doses as low as 1 Gy might have implications for both radiotherapy patients and astronauts on long-term space missions.

However, there are two sides of the coin. Currently, it is accepted that radiotherapy not only stimulates but can also activate the immune system turning the tumor into an *in situ* personalized vaccine (75). Diegeler and Hellweg emphasize the intercellular communication between tumor cells and immune cells after exposure to different radiation types and sustain our observation that the level of expression of cytokines, which modulate the immune cell behavior, is LET dependent (76). Carbon ions were also shown to induce anti-tumor immune response in a murine model (77). Another study examining five human cancer cell lines showed that comparable levels of high mobility group box 1, which plays an important role in activating anti-tumor immunity, were detected after irradiation with equitoxic doses of X-rays and carbon ions, meaning that a lower dose of carbon ions was needed to achieve the same effect (78). These results suggest that carbon ion therapy might activate the immune system to a greater extent than conventional radiotherapy, even when equivalent doses are used. Accumulating evidence demonstrates positive modulation of immune cells by radiation increasing their anti-tumor activity, however, there have also been reports of opposite effect of radiation inhibiting effective anti-tumor responses of immune cells (67). Therefore, further understanding of the effect of different radiation types on cytokine production by the immune cells is crucial for designing new therapeutic approaches combining radiation and immunotherapies.

Another important aspect of the transcriptional response to ionizing radiation (47, 54, 79) and other genotoxic agents (80–84) is alternative splicing and transcription. Exposure to low and moderate doses of low-LET ionizing radiation initiates alternative splicing and transcription of a large number of genes (47, 54, 79). In the present study, we also assessed the induction of alternative transcription and splicing by high- and low-LET radiation and observed a more pronounced response after exposure to heavy ions, especially carbon ions. A proteome-wide study in mouse embryonic fibroblasts exposed to carbon ions revealed significant changes in RNA metabolic processes, including RNA splicing (85). The exons most extensively regulated in response to X-ray exposure were not the most regulated after heavy ions exposure, suggesting specificity in response. Although it is not possible to draw any definite conclusions on the biological relevance of this observation from the microarray data, it is tempting to further study the role of alternatively transcribed/spliced genes in response to different radiation types. In a recent study exposure to UV was shown to trigger a shift from protein-coding mRNA of the *ASCC3* gene, which was alternatively spliced in response to heavy ions exposure in our set up, to a shorter non-coding isoform incorporating an alternative last exon. This RNA isoform, rather than the encoded protein, is critical for the eventual recovery of transcription (86). The non-coding ASCC3 isoform, in fact, counteracts the function of the protein-coding isoform and has an opposite effect on transcription recovery after UV-induced DNA damage (86).

Defects in DNA repair mechanisms often result in increased radiosensitivity of cells (87–91). Studies aiming at establishing an assay for predicting radiosensitivity focused on colony-forming assays (92, 93) or the measurement of DNA DSBs repair efficiency by means of the comet assay (94–96) or the γH2AX assay (96, 97). However, no single DNA damage-based assay proved to be capable of discriminating the full range of cellular radiosensitivity (98). A possible explanation is that radiosensitivity can also be associated with differences in cell cycle and apoptosis pathways regulation (99, 100). In this regard, transcriptional changes, which allow focusing on several instead of isolated cellular aspects, were suggested to be a promising predictive parameter for radiosensitivity (96, 101). Greve and co-authors identified a set of 67 differentially expressed genes in peripheral blood lymphocytes exposed to 5 Gy of γ-rays, which allowed distinguishing between the group of severely radiosensitive and non-radiosensitive breast, head and neck carcinoma patients (96). Rieger and co-workers used microarray gene expression profiling in lymphoblastoid cells derived from a diverse group of cancer patients with acute radiation toxicity. A set of 24 genes predicted radiation toxicity in 9 of 14 patients with no false positives among 43 controls (102).

In our study, we integrated the two approaches mentioned above, DNA DSB repair efficiency and transcriptional changes, based on the data of four donors after exposure of PBMCs to iron ions. It is important to mention that all the subjects involved in this study were apparently healthy, without any known abnormal variations in radiosensitivity. We aimed at exploring whether differences in gene expression can reflect the efficiency and kinetics of DSB repair measured by γH2AX assay. Although we did not find any significant differences in the repair efficiency between the four donors, the donor that showed the lowest repair rate, also displayed a distinct DNA damage repair gene expression profile after radiation exposure. This might imply that this individual is more radiosensitive compared to the other three donors. Interestingly, a recent study comparing transcriptional response of radiosensitive and radioresistant immortalized B-lymphocytes also showed a greater and prolonged response of p53-regulated genes in radiosensitive cells after exposure to 2 Gy of γ-rays (103). A similar approach of combining γH2AX with transcriptomics data was used for biodosimetry purposes in mice injected with ^137^Cs and proved to be more efficient than any of these methods alone (104).

Our study has limitations. First, the number of samples used for microarray experiments is limited to 4 to 10 per experimental condition, which might not be sufficient to draw definite conclusions at this stage. Moreover, gene expression changes in response to irradiation are very dynamic, thus the choice of the dose and time point is critically important for correct interpretation of the results. Our microarray study included only one time point and one dose, therefore direct translation of our results to radiotherapy, where higher doses are used, or space flights, during which the total doses and dose rates are lower, should be done cautiously. Nevertheless, several studies addressing the effect of dose rate on gene expression were previously performed in total body irradiated mice (105) and *ex vivo* irradiated human blood (3.1 mGy/min *vs* 1.03 Gy/min) (106). Overall, these studies showed that a significant number of genes responded similarly to low dose rate and acute exposures. Transcriptional response observed in blood samples obtained from radiotherapy patients undergoing total body irradiation was also in good accordance with the results obtained in *in vitro* studies (107, 108). Therefore, we believe that as our findings are in line with previously published results in other experimental models, they can serve as a solid basis for further studies. Second, at this stage our study describes the response to different radiation types only at transcriptional level, therefore the biological importance of our observations remains to be investigated. Finally, only 4 donors were considered. Although this study is small in scale, our results could be of interest for assessing the DNA repair efficiency and overall response to radiation in long-term space missions crew members and, potentially, radiotherapy patients. Moreover, gene expression measurements are more straight-forward and are technically less affected by such factors as radiation type compared to the γH2AX assay. At the same time, measuring gene expression for radiosensitivity assessment allows having a broader look at the cause of radiosensitivity as virtually any gene can be included in the assay. In conclusion, we have shown that both low- and high-LET irradiation induce similar transcriptional pathways, albeit with variable amplitude and timing, but that high-LET radiation also elicits specific and more persistent transcriptional events that may exacerbate the carcinogenic potential or, on the other hand, induce immune response against tumor cells. Our results imply that more detailed investigations of transcriptional response could bring new insight into differential normal tissue responses to high- and low-LET radiation and might have implications for the development of particle therapy treatment and radiation protection.

## Data Availability Statement

The original contributions presented in the study are included in the article/**Supplementary Material**. Further inquiries can be directed to the corresponding author.

## Ethics Statement

The studies involving human participants were reviewed and approved by SCK CEN Ethics Committee. The patients/participants provided their written informed consent to participate in this study.

## Author Contributions

EM, KT, AJ, and AM performed experiments. NA directed heavy ion irradiations at GSI. EM and RQ analyzed data. EM, KT, MM, SB, and RQ designed the study. MM, WV, SB, and RQ directed the work. EM and RQ wrote the manuscript. All authors contributed to the article and approved the submitted version.

## Funding

This work was supported by ESA PRODEX contracts (PEA 4000130301 and PEA 4000109861), the University of Antwerp (BOF/29267), by the ESA IBER-10 program project “GYMBRASS” (AO-10-IBER-26) and a grant from the Belgian FPS Economy, S.M.E.s, Self-employed and Energy (Belgian Royal Decree of February 23 2018, Article. 3. §4. 3° Project 19). EM is the recipient of a joint SCK CEN/UGent PhD scholarship. WV is member of the Research Excellence Consortium μNEURO at the University of Antwerp.

## Conflict of Interest

The authors declare that the research was conducted in the absence of any commercial or financial relationships that could be construed as a potential conflict of interest.

## Publisher’s Note

All claims expressed in this article are solely those of the authors and do not necessarily represent those of their affiliated organizations, or those of the publisher, the editors and the reviewers. Any product that may be evaluated in this article, or claim that may be made by its manufacturer, is not guaranteed or endorsed by the publisher.

## Glossary

ASCC3: activating signal cointegrator 1 complex subunit 3
ASTN2: astrotactin 2
ATM: ataxia telangiectasia mutated
ATR: ataxia telangiectasia and Rad3-related
cDNA: complementary deoxyribonucleic acid
cRNA: complementary ribonucleic acid
DDB2: DNA damage binding protein 2
DNA: deoxyribonucleic acid
DSB: double-strand break
EDTA: ethylenediaminetetraacetic acid
FC: fold-change
FDR: false discovery rate
FDXR: ferredoxin reductase
FITC: fluorescein isothiocyanate
GADD45A: growth arrest and DNA damage inducible alpha
GCR: galactic cosmic rays
GO: gene ontology
GSEA: gene set enrichment analysis
HIST2H3A: histone cluster 2 H3a
HIST2H3PS2: histone cluster 2 H3 pseudogene 2
HIST2H3C: histone cluster 2 H3c
HIST2H3D: histone cluster 2 H3d
HLA-A: major histocompatibility complex class I A
HLA-B: major histocompatibility complex class I B
HLA-H: major histocompatibility complex class I H (pseudogene)
HLA-DMB: major histocompatibility complex class II DM beta
HPRT1: hypoxanthine phosphoribosyltransferase 1
HZE: high (Z) atomic number and energy
IL-1: interleukin-1
IL-2: interleukin-2
IL-6: interleukin-6
JAK: Janus kinase
LET: linear energy transfer
MAMDC4: MAM domain containing 4
MCP-1: monocyte chemoattractant protein-1
MRE11A: meiotic recombination 11 homolog A
mRNA: messenger ribonucleic acid
mTORC1: mammalian target of rapamycin complex 1
NDUFAF6: NADH : Ubiquinone oxidoreductase complex assembly factor 6
NES: normalized enrichment score
NF-κB: nuclear factor κB
PBMC: peripheral blood mononuclear cell
PCNA: proliferating cell nuclear antigen
PGK1: phosphoglycerate kinase 1
qRT-PCR: quantitative reverse transcription polymerase chain reaction
RAD51D: DNA repair protein RAD51 homolog D
RBE: relative biological effectiveness
RBM14: RNA binding motif protein 14
RMA: robust multichip analysis
RNA: ribonucleic acid
RPS27L: ribosomal protein S27 like
RRHO: rank-rank hypergeometric overlap
SPE: solar particle event
STAT3: signal transducer and activator of transcription 3
STAT5: signal transducer and activator of transcription 5
TNF-α: tumor necrosis factor alpha
TP53: tumor protein 53
UV: ultra violet
VWCE: Von Willebrand factor C and EGF domains
γH2AX: phosphorylated histone subtype H2A isoform X.
