# Supplementary figures and images for: High-LET Carbon and Iron Ions Elicit a Prolonged and Amplified p53 Signaling and Inflammatory Response Compared to low-LET X-Rays in Human Peripheral Blood Mononuclear Cells
Source: Front Oncol. 2021 Nov 23;11:768493. doi: 10.3389/fonc.2021.768493 (PMC8649625; doi:10.3389/fonc.2021.768493)

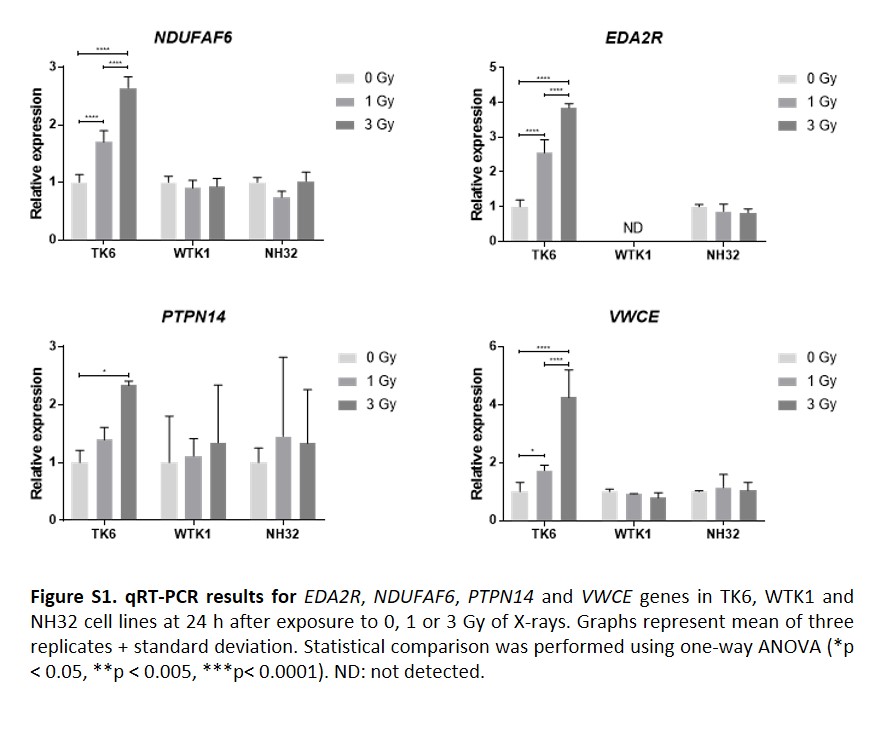

Supplement: Supplementary file 1 [file Image_1.jpeg]
