# Supplementary material for: High-LET Carbon and Iron Ions Elicit a Prolonged and Amplified p53 Signaling and Inflammatory Response Compared to low-LET X-Rays in Human Peripheral Blood Mononuclear Cells
Source: Front Oncol. 2021 Nov 23;11:768493. doi: 10.3389/fonc.2021.768493 (PMC8649625; doi:10.3389/fonc.2021.768493)
Supplement: Supplementary file 2 [file Table_1.docx]

| **Gene** | **Forward primer (5’ 🡪 3’)** | **Reverse primer (5’ 🡪 3’)** |
| --- | --- | --- |
| *ASTN2* | TCTGCAGCATAAGAAAGTGGATGA | CAATCAAGTTGGCTTCATCCCTG |
| *EDA2R* | CTTGCCCAGTTGTTCTCTCT | GGAGAATCAATCCTCTGGTG |
| *PTPN14* | GCAAGCTGATTTTGGAGACT | TTGTGTTCTTGGGCTACCTT |
| *FDXR* | GCAAGTGGCCTTCACCATTAAG | CCTTGATCTTGTCCTGGAGACC |
| *VWCE* | GACAGGCTGCTCTCTTGAC | AGTCTGTCCTCTTGCAGCTC |
| *HPRT1* | TCAGGCAGTATAATCCAAAGATGGT | AGTCTGGCTTATATCCAACACTTCG |
| *GADD45A* | CTGCGAGAACGACATCAAC | AGCGTCGGTCTCCAAGAG |
| *NDUFAF6* | AAAGAGAGACTGGAGCCACCT | GAGGAATGTGAAATGCTGATTGGC |
| *RPS27L* | GAGATTTACTACATCCGTCCTTGG | GAACCACTGTCTGAGCATGG |
| *PCNA* | GCACTGAGGTACCTGAACTT | TCTTCATCCTCGATCTTGGG |
| *MAMDC4* | TGGATGACGTGGAGTATCTG | CAGGAGCATGAGCAATAGGA |
| *PGK1* | CAAGAAGTATGCTGAGGCTGTCA | CAAATACCCCCACAGGACCAT |
